# Aortic stiffness and central hemodynamics in treatment-naïve HIV infection: a cross-sectional study

**DOI:** 10.1186/s12872-020-01722-8

**Published:** 2020-10-07

**Authors:** Pedro Martínez-Ayala, Guillermo Adrián Alanis-Sánchez, Luz Alicia González-Hernández, Monserrat Álvarez-Zavala, Rodolfo Ismael Cabrera-Silva, Jaime Federico Andrade-Villanueva, Karina Sánchez-Reyes, Moisés Ramos-Solano, Diego Alberto Castañeda-Zaragoza, David Cardona-Müller, Sylvia Totsuka-Sutto, Ernesto Cardona-Muñoz, Carlos G. Ramos-Becerra

**Affiliations:** 1 HIV Unit Department, University Hospital “Fray Antonio Alcalde”, Universidad de Guadalajara, Guadalajara, Mexico; 2 Arterial Stiffness Laboratory, Department of Physiology, Universidad de Guadalajara, Sierra Mojada 950, Building Q, Ground Floor, District Independencia, 44340 Guadalajara, Jalisco Mexico; 3 HIV and Immunodeficiencies Research Institute, Clinical Medicine Department, CUCS-Universidad de Guadalajara, Guadalajara, Mexico

**Keywords:** Arterial stiffness, HIV infection, Arteriosclerosis, Pulse wave velocity, Cardiovascular risk, Chronic inflammation, Central pulse pressure

## Abstract

**Background:**

Human immunodeficiency virus (HIV) infection is associated with a greater risk of cardiovascular disease (CVD). HIV infection causes a chronic inflammatory state and increases oxidative stress which can cause endothelial dysfunction and arterial stiffness. Aortic stiffness measured by carotid femoral-pulse wave velocity (cfPWV) and central hemodynamics are independent cardiovascular risk factors and have the prognostic ability for CVD. We assessed cfPWV and central hemodynamics in young individuals with recent HIV infection diagnosis and without antiretroviral therapy. We hypothesized that individuals living with HIV would present greater cfPWV and central hemodynamics (central systolic blood pressure and pulse pressure) compared to uninfected controls.

**Methods:**

We recruited 51 treatment-*naïve* individuals living with HIV (HIV(+)) without previous CVD and 51 age- and sex-matched controls (HIV negative (−)). We evaluated traditional CVD risk factors including metabolic profile, blood pressure (BP), smoking, HIV viral load, and CD4^+^ T-cells count. Arterial stiffness and central hemodynamics were evaluated by cfPWV, central systolic BP, and central pulse pressure (cPP) via applanation tonometry.

**Results:**

HIV(+) individuals presented a greater prevalence of smoking, reduced high-density lipoprotein cholesterol, and body mass index. 65.9% of HIV(+) individuals exhibited lymphocyte CD4^+^ T-cells count < 500 cells/μL. There was no difference in brachial or central BP between groups; however, HIV(+) individuals showed significantly lower cPP. We observed a greater cfPWV (mean difference = 0.5 m/s; *p* < 0.01) in HIV(+) compared to controls, even after adjusting for heart rate, mean arterial pressure and smoking.

**Conclusion:**

In the early stages of infection, non-treated HIV individuals present a greater prevalence of traditional CVD risk factors, arterial stiffness, and normal or in some cases central hemodynamics.

## Background

Cardiovascular disease (CVD) is one of the most common causes of death among individuals living with human immunodeficiency virus (HIV), with greater risk for myocardial infarction [1], ischemic stroke [2], and heart failure [3]. Furthermore, an accelerated rate of arterial stiffening has been reported after HIV infection, possibly due to the acute [4] and chronic inflammatory response [5], lipid disorders [6, 7], oxidative stress [8], and the adverse effects of some antiretroviral therapies (ART) [9]. The complex associations between chronic infection, inflammation, and endothelial function have long been studied, but the underlying mechanisms by which HIV infection per se increases the risk for CVD are not completely understood. HIV is capable of penetrating endothelial cells and is able to initiate inflammatory and biochemical intracellular reactions in the coronary arteries [10], the cerebral vasculature [11], and the aortic wall [12]. Moreover, aortic stiffness is associated with coronary artery disease [13, 14]; and it is an independent predictor of coronary events [15]. However, there are contradictory findings on aortic stiffness measured by carotid-femoral pulse wave velocity (cfPWV) in treatment-*naïve* individuals living with HIV; with some studies showing similar [16, 17] and others increased cfPWV, compared to controls [18, 19].

Identification of subclinical changes in the cardiovascular (CV) system, such as arterial stiffness and central hemodynamic assessment, is essential for a more accurate CV risk classification. Numerous studies have shown that arterial stiffness measured by cfPWV is an independent risk factor for CV events [20] and all-cause mortality [21, 22]. In addition, central hemodynamic assessment via applanation tonometry (central systolic blood pressure [cSBP] and central pulse pressure [cPP]), better reflect the load imposed on the left ventricle than brachial blood pressure (BP) [23]. The non-invasive central hemodynamic assessment has been reported to have a stronger relationship to organ and vascular damage, as well as to better predict CV events compared to peripheral pressures [24, 25]. Since people living with HIV exhibit a greater prevalence of hypertension [26] and risk of CVD compared to uninfected adults [27], it makes sense to assess central hemodynamics to detect early changes and provide treatment in a timely manner.

It has been reported that ART, specially protease inhibitors (PI), deteriorate arterial function [28]. Unfortunately, a significant amount of available literature looking at cfPWV in HIV combines both individuals on ART and treatment-*naïve* [29–31]. As a result, the impact of HIV infection per se on arterial function is not clear. For this reason, the objective of this study was to non-invasively assess and compare arterial stiffness and central hemodynamic in non-previously treated (treatment-*naïve*) individuals living with HIV versus people without HIV.

## Material and methods

### Study population

Between January 2015 and August 2019, individuals living with HIV were enrolled from the “Antiguo Hospital Civil de Guadalajara” in Guadalajara, Mexico. The study complied with the Declaration of Helsinki and was approved by the ethics committee of the Hospital Civil Fray Antonio Alcalde. Informed consent was obtained from every participant. At study entry, participants’ past medical history and demographic information was obtained by a questionnaire. Inclusion criteria for individuals living with HIV included: a) Patients with 18 years of age or older with confirmed HIV infection and no previous ART, b) Absence of current or previous rheumatological or neoplastic disease or CVD; c) Without opportunistic infections at the time of enrolment. Our control group was a group of healthy volunteers who were paired by age and sex. HIV infection was ruled out by a rapid serological test (Architect HIV Ag/Ab reagent kit, Abbot). In addition, lab tests and medical interrogation were performed to rule out metabolic alterations (e.g. diabetes mellitus or dyslipidaemia) or medical history of cardiovascular disease, in which case were excluded as controls. Inclusion criteria for the control group included: a) Negative HIV serological test, b) No previous cardiovascular or metabolic or rheumatological disease. We aimed to isolate the effect of HIV on arterial stiffness by choosing patients living with HIV without any other comorbidities and comparing them with healthy controls; however, some non-controlled factors were different between the population studied, such as tobacco use and sedentary lifestyle.

### Arterial stiffness

Arterial stiffness was measured by cfPWV as described previously [32] by applanation tonometry (PulsePen, Diatechne, Milan, Italy). cfPWV was calculated as the time delay between the arrival of the pulse wave at the carotid and the femoral artery, divided by the tape-measured distance between carotid and femoral arteries, minus the carotid-to-sternal notch distance. All measurements were performed by a single trained technician in a temperature-controlled room. The participants rested in a supine position for 15 min before the assessment and were instructed to abstain from smoking, alcoholic, or caffeinated beverages 24 h before the evaluation. Two consecutive measurements were performed, and the average cfPWV was used if the difference was lower than 0.5 m/s. Otherwise, a third measurement was obtained, and the median of the three measurements was used for analysis [33]. cSBP was estimated by applanation tonometry on the right carotid artery and calibrated with brachial diastolic blood pressure (pDBP) and mean arterial pressure (MAP) obtained by an automated sphygmomanometer (Omron HEM-907XL). MAP was calculated as MAP = pDBP + peripheral pulse pressure (pPP)ˑ0.33. cPP was determined as cPP = cSBP - pDBP.

### HIV variables and serum lipids

A venous blood sample was obtained from the antecubital vein after 8-h fasting. CD4^+^ T-cells count was performed by flow cytometry (FACScalibur System, Becton Dickinson) and HIV-1 viral load with real-time polymerase chain reaction with retro transcription (Cobas AmpliPrep/Cobas Taqman, Roche Diagnostics). Serum lipids, including total cholesterol (TC), high-density lipoprotein cholesterol (HDL-c), low-density lipoprotein cholesterol (LDL-c), and triglycerides (TG) were determined by colorimetric quantification (AU5800 autoanalyzer, Coulter Beckman, USA). Plasma glucose was determined by photometry (AU5800 autoanalyzer, Coulter Beckman, USA).

### Statistical analyses

Values are presented as mean and standard deviation (SD) or median and interquartile range (IQR), depending on the data distribution. Continuous data were compared using unpaired t-test or Mann-Whitney’s test if normally or not normally distributed, respectively. A Chi-square test was used to compared categorical variables. A generalized linear model was used to adjust cfPWV for age, sex, heart rate (HR), MAP, and body mass index (BMI), which affect and could confound PWV values [34]. Statistical analysis was performed using SPSS v.24 (IBM Chicago, IL), and for graphical representation, we used GraphPad Prism version 6.0 (Graph Pad Software, San Diego, California, USA). We calculated the sample size to detect a 0.8 m/s difference and standard deviation of 1.01 m/s, which resulted in 35 individuals in each group, at an alpha of 0.05 and a power of 90%. This calculation was based on the study conducted by Schillaci et al. [18]. The power calculation was performed using GPower 3.1.9.2 [35].. A two-sided *p*-value of < 0.05 was considered significant.

## Results

**Fig. 1:**
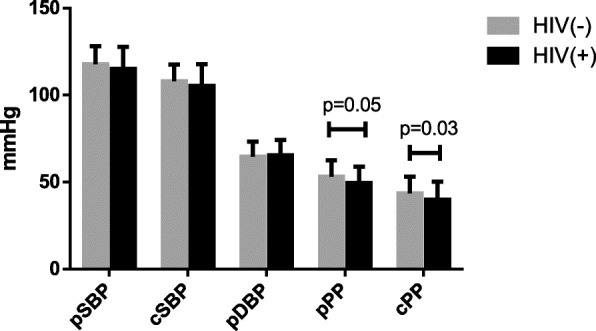
Peripheral and central hemodynamic parameters in people living with HIV(+) and HIV(−) controls. pSBP, peripheral systolic blood pressure; cSBP, central systolic blood pressure; pDBP, peripheral diastolic blood pressure; pPP, peripheral pulse pressure; cPP, central pulse pressure

We recruited 102 participants; 51 treatment-*naïve* individuals living with HIV(+) and 51 HIV (−) participants. HIV(+) individuals did not show evidence of opportunistic infections on the day of the assessment. Serum lipids and immune assessment were obtained from 51 individuals in the HIV(+) group and from 35 individuals in the control group. Clinical characteristics and hemodynamic values are shown in Table 1. There were no significant group differences regarding age, TG, or LDL-c. We observed a significantly higher prevalence of smoking, greater HR (*p* < 0.05), and lower body mass index (BMI) (*p <* 0.05) in HIV(+). We also found a tendency for lower TC (*p* = 0.08) but significantly lower HDL-c in the HIV(+) group compared to HIV(−). Regarding the immunosuppression state, 65.9% of HIV(+) presented CD4^+^ T-cells count < 500 cells/μL. We did not observe differences in peripheral (pSBP, MAP, and pDBP) or cSBP between groups. In the HIV(+) group, pPP showed a tendency to be lower, and cPP was significantly reduced compared to the HIV(−) group (Fig. 1). Lastly, the HIV(+) group exhibited greater cfPWV (mean difference = 0.5 m/s; 95% CI 0.26 to 0.86) compared to the uninfected group, even after adjustment for MAP, HR and current smoking (Table 2).

**Table 1:** Demographic, hemodynamic, metabolic and immune characteristics of the study groups

	HIV(−) (*n* = 51)	HIV(+) (*n* = 51)	*P* value
Age, years	31.9 ± 10.2	33.4 ± 9.9	0.44
Male sex, n (%)	45 (86)	45 (90)	0.56
Weight, kg	74 ± 13	66 ± 10	**< 0.01**
Cigarette smoking, n (%)	12 (23.5)	32 (62.7)	**< 0.01**
BMI, kg/m^2^	24.8 ± 3.3	23.2 ± 4.0	**0.04**
**Hemodynamic**			
pSBP, mmHg	117.8 ± 10.3	115.1 ± 12.9	0.24
pDBP, mmHg	64.5 ± 8.7	65.4 ± 8.8	0.59
MAP, mmHg	82.7 ± 8.3	82.3 ± 8.8	0.82
HR, bpm	65.7 ± 11.9	71.2 ± 13.7	**0.03**
pPP, mmHg	53.2 ± 9.4	49.6 ± 9.2	0.05
**Metabolic profile**			
TC, mmol/L	4.1 (3.5 to 4.7)	3.7 (3.1 to 4.4)	0.05
LDL-c, mmol/L	2.5 (1.9 to 2.9)	2.2 (1.8 to 2.7)	0.29
HDL-c, mmol/L	1.1 (1.0 to 1.3)	0.8 (0.7 to 0.9)	**< 0.01**
TG, mmol/L	1.2 (0.9 to 1.6)	1.4 (0.9 to 2.1)	0.25
Glucose, mmol/L	5.0 (4.7 to 5.3)	4.7 (4.4 to 5.2)	**0.04**
**Immune profile**			
CD4^+^ T, cells/μL	–	496 ± 298	
Viral load, copies/mL	–	70,250 (1173 to 2′279,000)	

Values are mean ± SD and median (IQR)

*BMI* body mass index, *pSBP* peripheral systolic blood pressure, *pDBP* peripheral diastolic blood pressure, *MAP* mean arterial pressure, *HR* heart rate, *pPP* peripheral pulse pressure, *TC* total cholesterol, *LDL-c* low-density lipoprotein cholesterol, *HDL-c* high-density lipoprotein cholesterol, *TG* triglycerides, *CD4*^*+*^ *T* CD4^+^ T-cells

**Table 2:** Central hemodynamics and arterial stiffness between groups

	HIV(−)	HIV(+)	*p*-value
cSBP, mmHg	107.6 ± 9.8	105.3 ± 12.4	0.23
cPP. mmHg	42.5 (36 to 52)	39 (31 to 47)	**0.03**
cfPWV, m/s	6.7 ± 1.0	7.3 ± 1.1	**0.04**
cfPWV, m/s (SE)^a^	6.8 (0.12)	7.4 (0.12)	**< 0.01**

Values are mean ± SD and median (IQR) unless otherwise indicated

*cSBP* central systolic blood pressure, *cPP* central pulse pressure, *cfPWV* carotid-femoral pulse wave velocity

^a^Adjusted for MAP, HR, BMI, and current smoking. SE, standard error

## Discussion

We found that treatment-*naïve* individuals living with HIV present greater arterial stiffness compared to age- and sex-matched controls. This difference remained significant after adjusting for HR, MAP, BMI, and current smoking. Despite a greater cfPWV, we paradoxically found a tendency for lower pPP and cPP in the group living with HIV. We replicated previous findings on the harmful effect of HIV infection itself on the arterial vasculature but in a Mexican population; it is crucial to establish the effect of chronic infectious diseases in different populations such as HIV, given that the immune activation has been reported – in some studies – to vary between ethnicities [36]. For example, compared to Caucasians, Hispanic individuals have been reported to present a greater prevalence of diabetes, insulin resistance [37], and greater average years of life lost after HIV infection [38]. Currently, it is unknown whether Latin-American individuals living with HIV could develop, to a greater degree, metabolic abnormalities secondary to inflammation and accelerate arteriosclerosis.

### Arterial stiffness

We found a greater cfPWV in treatment-*naïve* individuals living with HIV compared to controls, which agrees with previous reports in non-treated HIV infection [19, 39, 40]; however, others have reported similar cfPWV compared to controls [31, 41, 42]. As in our study, Schillaci et al. [43] found, besides increased cfPWV, lower BMI, and HDL-c in individuals living with HIV without ART. A greater aortic stiffness, in our studied population, could be a combination of functional and structural changes in the arterial wall. Arterial stiffness is a complex phenomenon where different factors intervene, such as endothelial dysfunction, smooth muscle vascular tone, and structural changes. One of the mechanisms that regulate endothelial function is nitric oxide (NO). NO produces vasodilation, inhibits inflammation, and prevents thrombosis [44]. Chronic inflammation and greater oxidative stress impair NO by reducing its bioavailability; both processes present during HIV infection [45, 46]. In animals, knock-out mice lacking superoxide dismutase (antioxidant) exhibited progressively greater PWV over time compared to the wild type mice [47]. In humans, a study showed that acute inflammation caused by typhoid vaccine administration resulted in endothelial dysfunction [8]. Moreover, in the early stages of HIV infection, it has been reported a decrease in glutathione [48] and total antioxidant capacity and increase in peroxidation potential [49] and gamma-glutamyl transpeptidase [18] – the latter associated with oxidative stress.

Another mechanism that may cause arterial structural changes is through matrix metalloproteinase (MMPs) dysregulation, which can which can degrade the collagen, elastin, laminin, and fibrillin within the arterial wall. Specifically, MMP-9 and MMP-2 – associated with vascular remodelling [50] and increased aortic PWV [51] – have been reported to present a 3.1 fold increase in HIV-infected macrophages and stimulated by HIV-derived proteins: envelope 120 and Tat [52, 53]... Although these endopeptidases were not assessed in our study, this mechanism could partially explain our findings.

Some current ART regimes have shown to have negative effects on the vasculature. A prospective study by Squillace et al. [54] reported that two PI regimens (atazanavir/ritonavir and lopinavir/ritonavir) increased pro-atherosclerotic chemokines, lymphocyte adhesion molecules and no improvement in arterial function after a 6–18 month follow-up.. On the other hand, the degree of immunosuppression has been associated with carotid arterial stiffness [55]. In our HIV cohort, we observed that 63.6% of individuals had a CD4^+^ T-cells count < 500 cells/μL, which may indirectly indicate a long-standing HIV infection before diagnosis; thus, a chronic and more detrimental effect on the vasculature.

### Central hemodynamics

We found similar brachial and central BP between groups but lower cPP and a tendency to decreased pPP in the individuals living with HIV) group compared to the HIV(−) group. The lower central and brachial PP may be caused by a combination of a slightly reduced cSBP and pSBP and higher pDBP. A slight decrease in cSBP, despite greater PWV, could be explained by peripheral vasodilation (small and medium-sized arteries), possibly due to the vasodilating effect of prostaglandins [56, 57] and inflammatory cytokines (i.e., interleukin 1beta) [58], through NO-independent mechanisms [59]. The vasodilation effect on peripheral reflection sites (e.g., arterial bifurcations) might cause a decreased reflection of the backward wave and a reduced contribution to cSBP [60]. Maloberti et al. [17] reported similar cPP between controls and individuals living with HIV receiving ART or *naïve* to it, except for those with chronic kidney disease; however, their cPP subgroup comparison was relatively small Likewise, Vlachopoulos et al. [41] reported similar cPP and cfPWV; however, they did observe a reduced cSBP in treatment-*naïve* HIV(+) compared to uninfected individuals.

### Lipid metabolism

Abnormal lipid metabolism after HIV infection is common and can be caused by the HIV infection itself, chronic inflammation, and ART (i.e., PI) [61, 62], which could lead to the development of metabolic syndrome (MetS). MetS is more prevalent in people living with HIV compared to non-infected, and further aggravated after ART [63]. Maloberti et al. [63] reported a higher prevalence of MetS in individuals with HIV receiving ART (19.4%) and treatment-*naïve* HIV individuals (13.8%) compared to controls (4.5%). In our study, we found a tendency for lower TC, significantly lower HDL-c, and similar TG levels in treatment-*naïve* HIV compared to uninfected controls. The majority of the individuals living with HIV in our study presented HDL-c < 1.04 mmol/L (78.2% vs. 21.8%) and TC < 5.2 mmol/L (60.8% vs. 39.2%) compared to the HIV(−) group, respectively [6]. Arterial stiffness has been associated with dyslipidaemia [64], in particular, hypertriglyceridemia and HDL-c play an essential role in the development of CVD. HDL-c provides atherogenic protection, prevents vascular inflammation [65] and oxidative stress [66]; thus, preserving endothelial function [67, 68].

Previous findings of the effect of HIV on arterial stiffness have not been consistent. This can be due to several causes, including a pooled comparison of individuals receiving and not receiving ART and different methodologies to assess arterial stiffness such as brachial-ankle PWV [69] and one-point PWV [70]. By exploring treatment-*naïve* individuals living with HIV, this study allowed us to exclude the potential negative effect of ART on the arterial system and evaluate the impact of HIV infection and traditional risk factors.

Our study has some limitations. Due to its cross-sectional design, we were unable to establish a causal relationship. The smoking history was statistically adjusted and we were not able to assess the presence of MetS due to the absence of basal waist measurements. In addition, factors such as unemployment, education, and socioeconomic status have been associated with arterial health, and where not measured in our study participants. Future studies should evaluate the behaviour of biomarkers of inflammation or vascular disease in response to different ART combinations to better understand their effects on the vasculature.

## Conclusion

Our study provides evidence that, in the early stages, non-treated HIV individuals living with HIV present greater arterial stiffness and prevalence of traditional CVD risk factors compared to non-infected controls. Paradoxically, central hemodynamics appears to remain unchanged or present a favourable profile.

## Data Availability

The datasets used or analyzed during the current study are available from the corresponding author on reasonable request.

## Abbreviations

HIV: Human immunodeficiency virus
CVD: Cardiovascular disease
cfPWV: Carotid-femoral pulse wave velocity
HIV(+): HIV positive
HIV(−): HIV negative
ART: Antiretroviral therapy
MAP: Mean arterial pressure
BP: Blood pressure
SBP: Systolic blood pressure
pDBP: Diastolic blood pressure
cPP: Central pulse pressure
pPP: Peripheral pulse pressure
HDL-c: High-density lipoprotein cholesterol
TC: Total cholesterol
LDL-c: Low-density lipoprotein cholesterol
TG: Triglycerides
HR: Heart rate
BMI: Body mass index
CI: Confidence interval
MetS: Metabolic syndrome
